# Identification of Known and Novel microRNAs and Their Targets in Peach (*Prunus persica*) Fruit by High-Throughput Sequencing

**DOI:** 10.1371/journal.pone.0159253

**Published:** 2016-07-28

**Authors:** Chunhua Zhang, Binbin Zhang, Ruijuan Ma, Mingliang Yu, Shaolei Guo, Lei Guo, Nicholas Kibet Korir

**Affiliations:** 1 Institute of Horticulture, Jiangsu Academy of Agricultural Sciences, Jiangsu Key Laboratory for Horticultural Crop Genetic Improvement, Nanjing, Jiangsu, China; 2 Department of Agricultural Science and Technology, Kenyatta University, Nairobi, Kenya; East Carolina University, UNITED STATES

## Abstract

MicroRNAs (miRNAs) are a group of non-coding RNAs that have functions in post-transcriptional gene regulation in plants. Although the most important economic component of peach trees (*Prunus persica*) is the fruit, not much is known about miRNAs in this organ. In this study, miRNAs and their targets were identified and characterized from libraries of small RNAs of peach fruit through Solexa based-sequencing and bioinformatics approaches. A total of 557 known peach miRNAs belonging to 34 miRNA families were identified, and some of these miRNAs were found to be highly conserved in at least four other plant species. Using the most current criteria for miRNA annotation, 275 putative novel miRNAs were predicted, and the sequencing frequencies of these novel miRNAs were less than those of the conserved miRNAs. In total, 3959 and 1614 target genes for 349 known and 193 novel miRNAs, respectively, were predicted with the criteria that a single target gene can be targeted by different miRNAs and that a single miRNA can also have a large number of target genes. Three targets were even found to be targeted by 13 novel miRNAs that contained the same complete miRNA sequence at different locations and had different scaffolds. The proteins predicted to be targeted by the miRNAs identified in this study encompass a wide range of transcription factors and are involved in many biological processes and pathways, including development, metabolism, stress responses and signal transduction. A total of 115 and 101 target genes were identified to be cleaved by 60 known miRNAs and 27 novel miRNAs through degradome sequencing, respectively. These miRNAs induce cleavage of their targets precisely at the position between nucleotides 10 and 11 of the miRNA sequences from the 5’ to the 3’ end. Thirty conserved miRNAs and 19 novel miRNAs exhibited differential expression profiles in the peach, and the expression patterns of some miRNAs appeared to be tissue- or developmental stage-specific. The findings of this study provide an important basis for the analysis of miRNAs, their targets and the functions of these targets in peach fruit.

## Introduction

MicroRNAs (miRNAs) are small non-coding RNA gene products often composed of approximately 22 nucleotides (nt). miRNAs are cleaved from 70- to 100-nt hairpin-shaped precursors (pre-miRNA) and are initially transcribed as slightly longer RNAs containing imperfect hairpins. From these initial RNAs, mature miRNAs are excised from the double-stranded region of the hairpin by Dicer-like enzymes [1–4]. Each mature miRNA is initially excised as a duplex comprising two ~22-nt RNAs: one of these is the mature miRNA, and the other, which is derived from the opposite arm of the hairpin, is known as the miRNA* [5–7]. The miRNA from this miRNA-miRNA* duplex is specifically loaded into the RNA-induced silencing complex, where it guides the post-transcriptional repression of its target mRNAs. In contrast, the miRNA* is postulated to undergo degradation [8–10].

miRNAs regulate their target mRNAs either by cleaving their target mRNAs within their binding regions or by repressing the successful translation of their target mRNAs [11,12]. Although their exact biological mechanism is not completely clear, miRNAs are found in various organisms and epigenetically function as negative regulators of gene expression [13,14]. Plant miRNAs exhibit a high degree of complementarity to their target genes with only 0- to 4-nt mismatches [1] and play critical roles in the regulation and control of gene expression [15] in various processes, including tissue morphogenesis [16], embryonic organ formation and separation [16], floral development [17], and stress responses [5,18]. Since 2002, when the first plant miRNA was discovered [19], a large number of miRNAs have been identified both experimentally and/or computationally in a variety of plant species, including beans [20], peanut [21], maize [8] and grapevine [6].

Peach fruit [*Prunus persica* (L.) Batsch] is a delicious and nutritious deciduous fruit crop grown widely in temperate regions of the world. The top five peach-producing countries are China, Italy, Spain, USA and Greece [20]. Since the peach genome was sequenced and its gene annotation was made publicly available on the Genome Database for Rosaceae (GDR), several reports on the identification of miRNAs in the peach leaf, winter buds, root, stems, and flower buds by high-throughput sequencing have been published [22–25], but there remains a dearth of comprehensive data on the characterization of miRNAs and their target genes in peach fruit. This gap exists despite the peach fruit being the most complex component with important economic value in peach trees. The aim of this study was to perform a comprehensive analysis of miRNAs from the fruit tissue using high-throughput sequencing combined with bioinformatics analysis and molecular experiments. The study found a total of 557 known and 275 novel miRNAs from the peach fruit small RNA (sRNA) library. We further predicted 3959 targets for 349 of the 557 known miRNAs and 1614 targets for 193 of the 275 novel miRNAs. A Gene Ontology (GO) and Kyoto Encyclopedia of Genes and Genomes (KEGG) analysis revealed that most of the identified target genes are involved in a large range of biological processes and function in various activities in addition to encoding numerous transcription factors. The quantitative reverse transcription-polymerase chain reaction (qRT-PCR) results indicated that a number of known miRNAs are widely expressed in the fruit and most phloem tissues of peach trees. The findings from this study add to our understanding of miRNAs in peach fruit through the identification of more miRNAs and their potential target genes. In particular, these data strengthen the foundation needed for the study of miRNAs and their functions in peach fruit and even in other species of Rosaceae.

## Materials and Methods

### Plant materials

Samples of the leaves, fruits and phloem were collected from six-year-old ‘Troubadour’ peach trees at five different stages of development grown under standard field conditions at the National Peach Germplasm Repository in Nanjing, China. The fifth leaf from the apex, the fruits, and the phloem on the same one-year-old fruiting shoot were sampled from the outer southern canopies of trees 35, 55, 75, 90, and 105 days after full bloom (DAFB). Nine ‘Troubadour’ trees were divided evenly into three plots. On each sampling date, one fruiting shoot was selected from each tree. Tissues of the same type from each plot were mixed together, and the resulting mixture was considered one replicate corresponding to a tissue sample from one of the three plots. All of the samples were immediately frozen in liquid nitrogen and stored at -80°C.

We confirm that this study did not involve any endangered or protected species. No specific permissions were required for the collection of peach materials from the National Peach Germplasm Repository in Nanjing, China, because our research team at the Institute of Horticulture constructed this National Peach Germplasm Repository in Nanjing, China, and we are responsible for the management of these peach trees.

### Total RNA extraction, construction of sRNA library and Solexa sequencing

The total RNA from the fruit samples was extracted using the Trizol reagent according to the manufacturer’s instructions and subsequently treated with DNase I (Takara Biotechnology Co., Ltd, Dalian, China) to remove contamination by genomic DNA. Equal amounts of total RNA from peach fruits at each of the different growing stages were then mixed and deep sequenced. The construction of the fruit sRNA library, cluster generation and high-throughput sequencing were performed by the Beijing Genomics Institute (BGI, Shenzhen, China) using the Illumina Solexa platform. sRNA fractions that were 18 to 30 nt in length were first purified from the low-molecular-weight RNA by excision from 15% denaturing polyacrylamide gels and then ligated to 3’ and 5’ adaptors using T4 RNA ligase according to the manufacturer’s instructions. The sRNAs ligated with 3’ and 5’ adaptors were purified and reverse transcribed to cDNA with the reverse transcription primers for 17 cycles according to the recommended protocol. The PCR products were then further purified and subjected to sequencing using the Illumina Genome Analyzer. The image files obtained from the analyzer were then evaluated and used to establish a large repertoire of digital data suitable for bioinformatics analysis.

### Identification of known and novel miRNAs in peach fruit

To identify known and novel miRNAs in peach fruit, low-quality sRNA reads were first filtered from the raw sequences obtained from the sequencing results. Several types of impurities, including sRNAs containing 3’ adaptor null reads, insert null reads, and 5’ adaptor contaminants, were removed from the remaining high-quality sRNA reads. The length distribution of the clean sRNAs was then summarized, and reads smaller than 18 nt or having Poly(A) tails were deleted. The remaining clean sRNA reads were aligned to repeat-associated RNAs as well as exons and introns of mRNA in GenBank (ftp://ftp.ncbi.nlm.nih.gov/genbank/) and Rfam (10.1) (http://rfam.janelia.org/) for annotation into different sRNA categories (rRNA, tRNA, snRNA, snoRNA, exons and introns, and the degraded fragments of mRNA) using the tag2repeat, overlap and BLASTn tools. The reads with a perfect match or one mismatch to these sRNA categories were removed, and all of the remaining clean sRNA reads were mapped to the peach genome using the SOAP (Short Oligonucleotide Alignment Program) tool to analyze their expression frequencies and locations on the scaffolds. These clean sRNA reads were further aligned against the miRNA precursors/mature miRNAs of all plants deposited in miRBase 18.0 using tag2 miRNA software to obtain the sequences and counts of known miRNAs that are found in the library of peach fruit sRNAs and are conserved in other plant species. sRNAs that did not map to any of the above-mentioned categories were termed unannotated sRNAs. These unannotated sRNAs were subsequently analyzed for the prediction of novel miRNAs using the program MIREAP (https://sourceforge.net/projects/mireap/) based on secondary structure predictions. The secondary structures of stem-loop hairpins were considered peach-specific miRNAs (novel miRNAs) only when they met all of the following criteria: miRNA sequence length between 18 and 25 nt; miRNA reference sequence length ranging from 20 to 23 nt; copy number of reference miRNAs of not more than 20 nt; maximum free energy for a miRNA precursor of -18 kcal/mol; space between miRNA and miRNA* of not more than 300 nt; miRNA and miRNA* base pairs of not less than 16 nt; miRNA and miRNA* bulge of at most 4 nt; maximum asymmetry of miRNA/miRNA* duplex of 4 nt; and flank sequence length of miRNA precursor of 20 nt.

### Prediction of target genes for known and novel miRNAs and GO and KEGG analyses

The criteria used for the prediction of miRNA targets followed those proposed by Allen et al. [26] and Schwab et al. [27] as follows: (1) at most four mismatches in the region of the miRNA/target duplex (a G-U match was considered 0.5 mismatches); (2) maximum of two adjacent mismatches in the complementary region of the miRNA and target; (3) no adjacent mismatches from the second to 12^th^ nucleotide in the complementary region of the miRNA and target (5' of miRNA); (4) no mismatches in the 10th and 11^th^ nucleotides of the miRNA and target complementary region; (5) maximum of 2.5 mismatches from the first to 12^th^ nucleotide of the miRNA and target complementary region (5' of miRNA); and (6) minimum free energy (MFE) of the miRNA/target duplex of not less than 75% of the MFE of the miRNA bound to its target. The putative miRNAs were first blasted against the peach genome database on the GDR, and the BLASTn hits that fulfilled the above-mentioned criteria were considered the predicted target genes. The Gene Ontology (GO) database (http://www.geneontology.org/) was then searched to annotate the putative genes involved in cellular components, biological processes, and molecular functions. All predicted target genes were mapped to GO terms in the GO database by counting the percentage of gene numbers for each term. We used a Bonferroni correction to obtain a corrected p-value. GO terms with a corrected p-value ≤ 0.05 were considered significantly enriched in the predicted target genes. The main pathways in which the target gene candidates are involved were then analyzed through the Kyoto Encyclopedia of Genes and Genomes (KEGG) methodology, which is the major public pathway-related database [28]. A KEGG analysis identifies metabolic pathways or signal transduction pathways significantly enriched with the candidate target genes compared with the entire reference gene background.

### Degradome sequencing and bioinformatic analysis

The total RNA of peach fruit was extracted using the Trizol reagent (Invitrogen, CA, USA) following the manufacturer’s recommended procedure. The total RNA quantity and purity were analyzed using a Bioanalyzer 2100 and RNA 6000 Nano LabChip Kit (Agilent, CA, USA) with an RIN number greater than 7.0. Approximately 20 μg of total RNA was used to prepare a degradome library. The method used for the construction of the degradome library followed the method reported by Ma et al. [29] with some modifications. (1) Approximately 150 ng of Poly(A)+ RNA was used as input RNA and annealed with Biotinylated Random Primers. (2) RNA fragments were captured by magnetic beads. (3) The captured fragments were then ligated to 3’ and 5’ adaptors using T4 RNA ligase according to the manufacturer’s instructions, and 5’ adaptors were only ligated to those RNAs containing 5’ monophosphates. (4) The sRNAs ligated with 3’ and 5’ adaptors were purified and reverse transcribed to cDNA with the reverse transcription primers for 15 cycles. (5) The final PCR product was purified, and the purified cDNA library was used for cluster generation with Illumina’s Cluster Station and then sequenced on an Illumina Hiseq 2500 instrument following the instruction provided for running the instrument. Raw sequencing reads were obtained using Illumina’s Pipeline v1.5 software following sequencing image analysis using the Pipeline Firecrest Module and base-calling with the Pipeline Bustard Module. The extracted sequencing reads were stored in a file and were then used in the standard data analysis. The GDR database and transcript sequences were used as the reference sequences. The CleaveLand3.0 software package was used for analyzing the sequencing data generated. The degradome reads were mapped to the appropriate transcriptome using appropriate thresholds. The mapped degradome data were summarized into a "degradome density file" using the 'CleaveLand3_map2dd.pl' script. Small RNA/mRNA alignments were then generated using 'targetfinder.pl'. The degradome density file was compared with the target prediction, and significant hits were obtained using the CleaveLand3_analysis.pl' script. "T—plots" of the targets were generated using the 'CleaveLand3_t—plotter.pl' script.

### Detection of expression levels of some known and novel miRNAs by qRT-PCR

For qRT-PCR analysis of miRNAs, sRNA was extracted from peach fruits, phloem, and leaves at different stages of development using the RNAiso for Small RNA extraction kit (Takara Biotechnology Co., Ltd., Dalian, China). Thereafter, the sRNA was treated with DNase I and reverse transcribed using a Transcript miRNA First-Strand cDNA Synthesis SuperMix Kit (TransGen Biotech Co., Ltd., Beijing, China) following the manufacturer’s instructions. All cDNA samples were diluted 1:10 with RNase-free water and stored at -80°C before use as templates in qRT-PCR. One of the specific primers used for the amplification of each miRNA through qRT-PCR was the sequence of each miRNA, and the other primer was a universal primer contained in the reverse transcription kit. *5S rRNA* was used as the reference gene. The primer sequences for each miRNA used for qRT-PCR are provided in S1 Table.

The qRT-PCR reaction was conducted on an Applied Biosystems 7500 Real-Time PCR System using a TransStart Top Green qRT-PCR SuperMix (TransGen Biotech Co., Ltd., Beijing, China) according to the manufacturer's instructions. The 20-μl qRT-PCR reaction volume contained 2.0 μl of diluted cDNA, 0.4 μl of each primer, 0.4 μl of passive reference dye, 10.0 μl of Supermix, and 6.8 μl of RNase-free water. The thermal cycling conditions were an initial polymerase activation step for 30 sec at 95°C followed by 40 cycles of 5 sec at 95°C for template denaturation and 34 sec at 60°C for annealing (fluorescence measurement). Stage 3 was 15 sec at 95°C, 1 min at 60°C and 15 sec at 95°C. Each assay was replicated three times. The raw fluorescence data obtained from the 7500 Real-Time PCR detection system were exported to Microsoft Excel software (Microsoft Corporation, USA), and the relative quantification expression levels of the genes were analyzed using the 2^-ΔΔCT^ method [30].

## Results

### Characteristics and sequence analysis of the sRNAs

**Fig 1:**
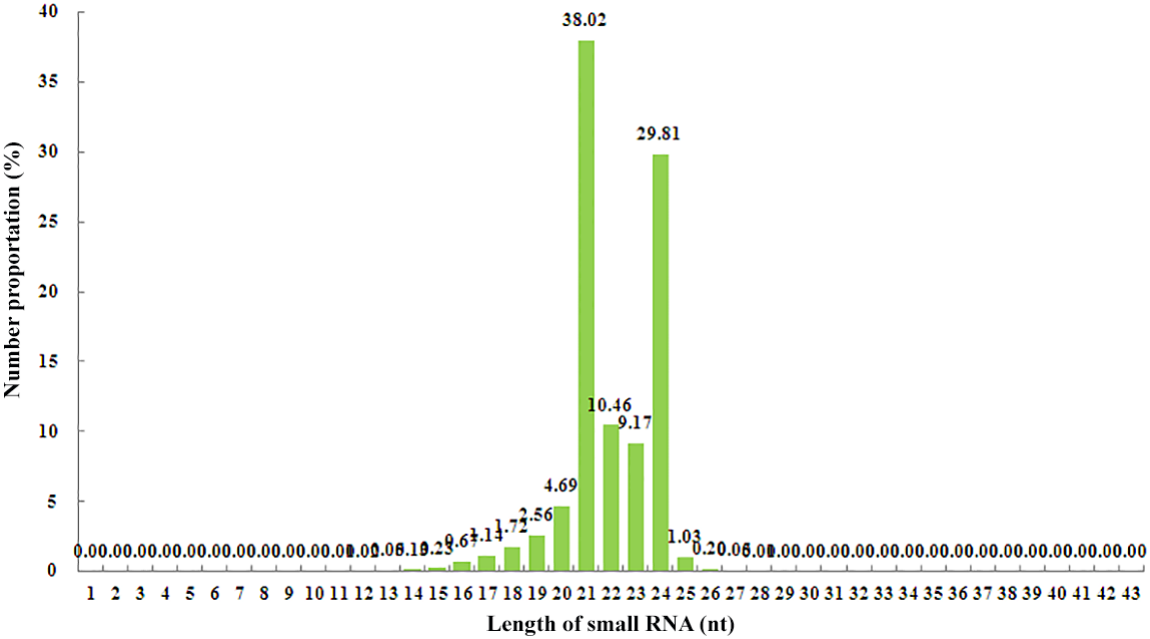
Length distribution of all small RNAs obtained by sequencing from a library of the small RNAs of peach fruit.

To identify known and novel miRNAs in peach fruit, a sRNA library was constructed using total RNA extracted from mixed peach fruits (cv. ‘Troubadour’) at different stages of development. After Solexa sequencing, a total of 17,186,803 raw reads were obtained (Table 1), and low-quality reads as well as other types of contaminant reads, such as those with 5’ adaptor contaminants (31,246) and without the insert tags (3,712) or 3’ primers (6,753), were removed, resulting in a total of 17,061,467 reads that were uploaded and subjected to a length distribution analysis. A statistical analysis of the length distribution revealed that the most frequently observed read length was 21 nt followed by 24 nt, and the proportions of the 17,061,467 reads with these lengths were 38.02 and 29.81%, respectively (Fig 1). Filtering off reads with poly(A) and reads that were less than 18 nt in length yielded a total of 16,675,173 clean reads (Table 1), and these were aligned against the peach genome using the SOAP tool to reveal their expression and distribution on the genome. The results indicated that 12,521,493 total reads and 2,440,952 unique reads, corresponding to 75.09% and 73.33% of the 16,675,173 (total) and 3,328,500 (unique) reads, respectively, were perfectly mapped to the peach genome. Furthermore, a statistical analysis of the first nucleotide in 18- to 25-nt sRNAs demonstrated that sRNAs starting with a uridine (U) at the 5’ end are more commonly observed in reads with lengths of 18 and 22–25 nt than other sRNAs of the same length starting with different nucleotides (Table 2).

**Table 1:** Summary of small RNA reads obtained by Illumina sequencing from a library of the small RNAs of peach fruit.

Type	Count	Percentage (%)
Total reads	17,186,803	
High quality	17,103,178	100.00
3’ adaptor null	6,753	0.04
Insert null	3,712	0.02
5’ adaptor contaminants	31,246	0.18
Smaller than 18 nt	386,179	2.26
Poly(A)	115	0.00
Clean reads	16,675,173	97.50

**Table 2:** Statistical analysis of first nucleotide of 18- to 25-nt small RNAs in a library of the small RNAs of peach fruit.

Length of sRNA	A	U	C	G
18	19,692	234,512	882	496
19	3,318	16,504	32,692	49,261
20	22,103	12,117	258,565	30,055
21	494,149	2,350,934	10,340	5,890
22	4,085	101,115	3,793	5,075
24	2,864	5,990	2,708	1,220
25	7,427	17,958	319	30,261

After a series of additional alignments against the peach genome on GenBank, Rfam (10.1), miRBase, repeat-associated RNA, and degraded fragments, the peach sRNAs were matched into different categories based on their biogenesis and annotation. The numbers of sRNA reads matched to each sRNA category in the above-mentioned databases at each step are summarized in Table 3. Among the 16,675,173 total reads, 249,923, 515,834, 264,105, 572,501, 2,342,951, 11,118, 3,394, and 737,196 sRNA reads matching the exon antisense, exon sense, intron antisense, intron sense, rRNA, snRNA, snoRNA, and tRNA categories, respectively, were discarded. The remaining unannotated sRNAs that failed to map to any of the above-mentioned categories (miRNA, rRNA, snRNA, snoRNA, tRNA, repeat, exon, and intron) and exhibited the greatest ratio (49.91%) of total reads were retained for the subsequent prediction of novel miRNAs. These results imply an abundance of miRNAs in the sRNA library of peach fruit.

**Table 3:** Summary of small RNA reads by annotation category in a library of the small RNAs of peach fruit.

Categories	Unique sRNAs	Percentage (%)	Redundant sRNAs	Percentage (%)
Exon antisense	81,691	2.45	249,923	1.50
Exon sense	146,581	4.40	515,834	3.09
Intron antisense	77,675	2.33	264,105	1.58
Intron sense	117,152	3.52	572,501	3.43
miRNA	39,245	1.18	3,654,996	21.92
rRNA	85,358	2.56	2,342,951	14.05
snRNA	2,608	0.08	11,118	0.07
snoRNA	858	0.03	3,394	0.02
tRNA	10,042	0.30	737,196	4.42
Unannotated RNA	2,767,290	83.14	8,323,155	49.91
Total	3,328,500	100.00	16,675,173	100

### Identification of known miRNAs in peach fruit

To identify known miRNAs in peach fruit, the candidate peach miRNA sequences were aligned with the newly released mature plant miRNAs in miRBase 18.0 [31] using miRAlign (BGI). The expression profiles of these miRNAs are presented in S2 Table. In total, 557 known miRNAs belonging to 34 miRNA families were identified in the library of peach fruit. Various miRNAs were found to be highly conserved with those from several other plant species. For example, miR156, miR160, miR162, miR164, miR166, miR167, miR169, miR172, miR396, and miR397 are perfectly identical to the corresponding miRNAs in three other plant species (*Arabidopsis thaliana*, *Populus trichocarpa*, and *Oryza sativa*) (S3 Table). miR164, miR166, and miR397 are very similar to the corresponding miRNAs in four plant species, including *Brassica napus* and *Vitis vinifera*.

Illumina sequencing offers a different method for estimating the expression levels of miRNA genes and can thus positively influence the ease of determining the enrichment of diverse miRNA families and even distinguishing among different members of a given family in one tissue or plant [32–34]. The abundance of members of different miRNA families also varied markedly in peach fruit. The most abundantly expressed miRNA family was miR156, which is represented by more than 1,910,151 reads (S2 Table), followed by miR166 with more than 39,453 reads. However, various miRNAs, such as miR391 and miR407, had only one read sequenced.

### Identification of novel miRNAs in peach fruit

**Fig 2:**
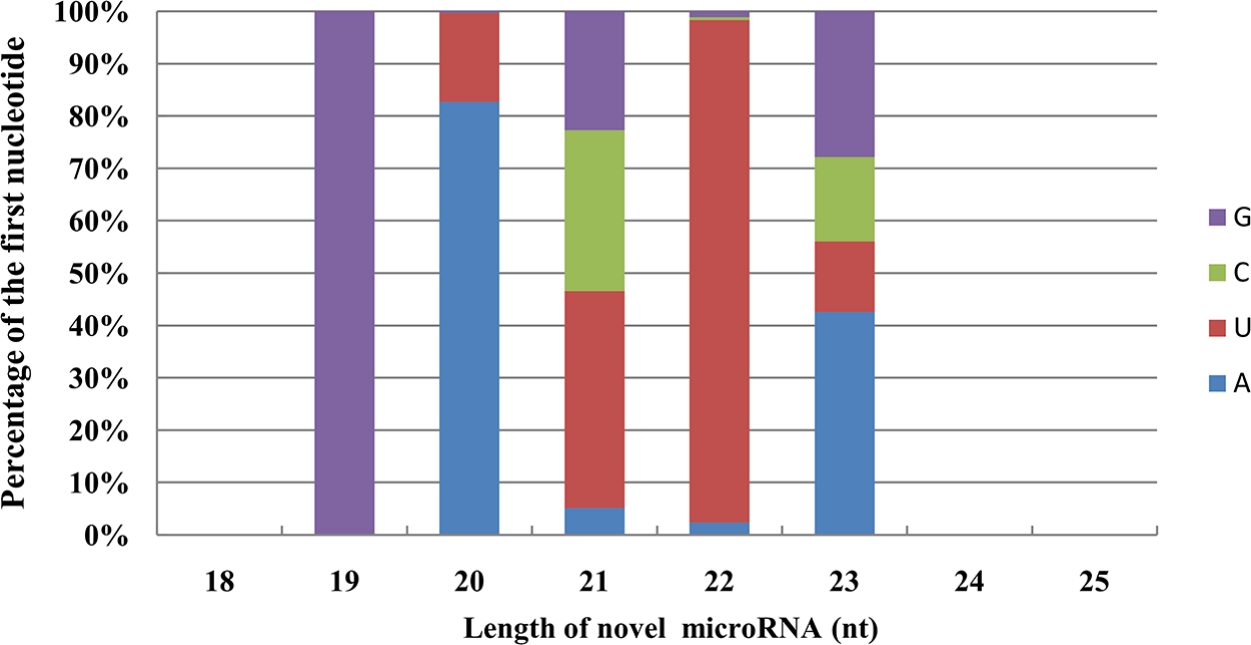
Proportion of first nucleotide bias within novel miRNAs of the same length in a library of the small RNAs of peach fruit.

**Fig 3:**
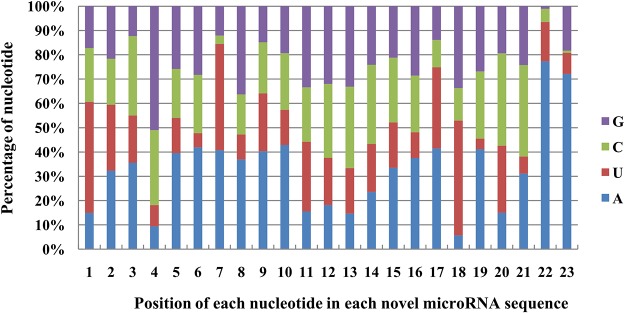
Proportion of nucleotide bias at each position within novel miRNAs in a library of the small RNAs of peach fruit.

After obtaining the known miRNAs, the remaining unannotated sequences from the peach fruit sRNA library were aligned against the peach genome database to predict novel miRNAs. Using MIREAP software and following a set criteria of miRNA annotations [35], 275 putative unique novel miRNAs were predicted (S4 Table) in the peach fruit sRNA library. The size distribution of these novel miRNAs ranged from 19 to 23 nt, with 21-nt-long novel Ppe-miRNAs being the most abundant (57.34%). The analysis of the first nucleotide of the novel miRNAs with the same length revealed that all of the 19-nt novel miRNAs started with G (82.70%), whereas the first nucleotide of the 20-nt novel miRNA candidates was A. The U nucleotide was dominant (96.00%) in the 22-nt putative novel miRNAs (Fig 2). The first nucleotide bias analysis revealed that U was the most frequently observed first nucleotide in the novel miRNAs of peach, with 23,730 (45.72%) of the 51,968 sequences in the fruit library having U as their first nucleotide (Fig 2). In addition, an analysis of the nucleotide bias at each position in each of these miRNAs revealed that novel miRNAs with a U at their 5’ ends presented the highest frequency among the 19- to 23-nt-long novel miRNAs (Fig 3). These findings are consistent with previous reports [36–38] that most of the miRNAs are 21 nt in length and start with a 5’ U, which is one of the classic features of miRNAs.

The sequencing frequency of miRNAs is thought to reflect the relative degree of miRNA abundance and is therefore recognized to represent miRNA expression profiles [39]. Although the 275 novel miRNAs were sequenced at varying frequencies ranging from 5 to 6973, a number of miRNAs dominated the novel miRNA library. The sequencing frequencies of the four most abundantly expressed miRNAs (m0187, m0100, m0189 and m0269) in this study were 6973, 6300, 5552, and 4520, respectively, suggesting that novel miRNAs might be unevenly expressed in peach fruit. Additionally, the expression frequency of these novel miRNAs was noticeably reduced compared with those observed for known miRNAs in the peach fruit sRNA library. Our MFE analysis of the peach fruit sRNA library using MIREAP revealed that the MFE of 275 putative novel miRNAs varied from -18.32 to -181.01 kcal/mol, with an average of -55.68 kcal/mol. The results obtained using the MIREAP tool also indicated that the putative pre-miRNAs of each miRNA varied from 74 to 365 nt in length.

### Prediction of target genes for known miRNAs in peach fruit and functional analysis

Because most miRNAs have near-perfect complementarity to their target genes, target prediction was conducted following the criteria specified by Allen et al. [26] and Schwab et al. [27]. Based on these rules, a total of 3,959 putative miRNA targets were predicted for 349 out of 557 known miRNAs from the peach fruit library (S5 Table). No target genes were predicted for 208 of the 557 known miRNAs, whereas the numbers of targets for each of the remaining 349 miRNAs ranged from one to 707. In all, 74 out of 349 known miRNAs (21.20%) only had one target gene. In contrast, the number of target genes for miR4993 was 707, comprising 17.86% of all the target genes in the peach fruit library. miR414 ranked second, with 8.69% of the total number of target genes.

The biological processes, cellular components and molecular functions of the miRNA target genes were summarized through a GO annotation analysis (S6 Table). The putative target genes of the miRNAs under study were found to be involved in a wide range of biological processes, with target genes responsible for cellular and metabolic processes exhibiting the highest cluster frequencies of up to 66.2% and 66.4%, respectively, among the 1491 genes annotated to the process ontology terms. Some of the predicted target genes of miRNAs in the category of biological process are also responsible for responses to environmental factors, such as absence of light (0.2%), gravity (0.3%), light intensity (0.5%), nutrient levels (0.8%), and various stimuli, including endogenous stimuli (7.4%), amino acid stimuli (0.1%), extracellular and external stimuli (1.6%), ethylene and hexose stimuli (0.4%), and carbohydrate stimuli (0.2%).

A total of 319 GO function terms were annotated for the putative target genes of miRNAs in the peach fruit sRNA library. Among these, the target genes with functions in binding and catalytic activity presented the highest cluster frequency, corresponding to 66.1% and 66.3%, respectively, of the 1713 genes annotated to the molecular function ontology terms (S6 Table). Various target genes were associated with transferase activity (25.6%), hydrolase activity (21.8%) and nucleotide binding (21.5%). The functions of other target genes had relatively lower percentages, including structural molecule activity (1.7%), signal transducer activity (2.9%), and UDP-glucosyltransferase activity (1.0%).

In addition, the annotation of 1412 genes to the component ontology terms revealed that the majority of target genes were located in the intracellular space (71.0%), organelles (65.9%) and cells (97.3%) (S6 Table). Various target genes were found to exist on the cell wall (2.6%), plastid envelope (3.5%), Golgi membrane (0.3%), replication fork (0.1%), and nucleoplasm (0.6%).

Based on a KEGG pathway analysis, a total of 3959 target genes in the peach fruit sRNA library were found to be involved in 248 different cellular pathways. These pathways include anthocyanin biosynthesis, flavone and flavonol biosynthesis, the biosynthesis of secondary metabolites and carotenoid biosynthesis (S7 Table). Most of the potential targets were members of different gene families, such as the homeobox leucine zipper protein (HD-ZIP) family, the auxin response factor family (ARF), the nucleobase cation symporter-1 (NCS1) family, the transparent testa 2 (TT2) family and the MYB domain protein family. A small percentage of targets corresponded to sulfur metabolism, peptidoglycan biosynthesis and mineral absorption.

### Predication of target genes for novel miRNAs in peach fruit

After searching the annotated set of filtered peach genes and removing any repeated candidates or others not meeting the criteria, a total of 1614 target genes for 193 out of the 275 novel miRNAs were revealed. No target gene was identified for the remaining 62 novel miRNAs. The number of predicted targets per miRNA varied from one to 457 (S8 Table); specifically, 109 out of the 193 miRNAs had two to 10 targets, whereas 19 miRNAs had more than 10 targets. m0246 had the highest number of targets at 457 targets. In all, 65 of the 193 novel miRNAs with targets only targeted one gene. It has been reported that in some cases, a single gene can be targeted by several miRNAs [40], and this finding was corroborated in this study. For example, ppa001561m was found to be targeted by m0028, m0103, m0184, and m0231. Additionally, our study revealed the following specific and interesting feature: 13 novel miRNAs (m0004, m0015, m0052, m0104, m0117, m0118, m0155, m0170, m0175, m0188, m0209, m0270, and m0272) had the same miRNA sequence and shared three target genes (ppa012437m, ppa013211m, and ppa017834m) at different locations (S4 and S8 Tables).

Consistent with earlier reports [41] on the essential roles of miRNAs in regulating a variety of biological processes in plants, the predicted targets of the novel miRNAs predicted in this work appear to play roles in diverse physiological processes (S8 Table). Most of the identified targets were transcription factors of different families that control plant development and phase change from vegetative growth to reproductive growth, and some examples include GRAS as a target of m0103 and m0028, MYC2 as a target of m0268 and WRKY as a target of m0265. The 11 target genes of m0233 have squamosal promoter-binding protein (SBP) domains, indicating that these genes are members of the SBP family that likely function as transcription factors involved in the control of early flower development.

Target genes encoding HD-ZIP proteins, F-box proteins, and DNA-binding proteins all had perfect or near-perfect complementary binding sites with some miRNAs identified in this study. A total of two out of five target genes of m0247 have been predicted to encode No Apical Meristem (NAM) proteins, which determine the positions of meristems and primordia. In addition to affecting plant development, several predicted targets of m0259 and m0025 encode disease-resistance protein RPM1, which functions in plant-pathogen interaction mechanisms (S8 Table). We also found that P-loop-containing nucleoside triphosphate hydrolases (P-loop NTPase domain superfamily) are potential targets of m0247, m0259 and m0128, indicating that these play roles in signal recognition. Despite these observations, the functions of a large number of the predicted targets remain unknown.

### Verification of targets of known and novel miRNA by degradome sequencing

To validate the targets of peach fruit miRNAs, degradome sequencing was performed in this study to detect the exact cleavage site of each miRNA to its target. After degradome library construction, sequencing and bioinformatics analysis using CleaveLand 3.0 (http://sites.psu.edu/axtell/software/cleaveland4/), a total of 115 and 101 genes were identified to be targeted by 60 known miRNA families and 27 novel miRNAs, respectively (S9 Table). These miRNAs induce cleavage of their targets precisely at the position between nucleotides 10 and 11 of the miRNA sequences from the 5’ to the 3’ end. Some validated targets and cleavage sites were displayed clearly in the form of ‘t- plots’, as shown in S9 Table. The analysis of these identified target genes revealed that a single target gene could be targeted and cleaved by several miRNA families and that a single miRNA family could target several different genes with different cleavage sites for each gene. For example, the miR156 family was verified to cleave five members of the squamosa promoter-binding protein-like (SPL) transcription factor family. Remarkably, the degradome sequencing results verified that four HD-ZIP III subfamily genes (HB8, HB14, HB15, and REV), which were predicted but had not been previously validated, were verified to be cleaved by the miR166 family of peach fruit at nucleotides 565, 574, 589, and 615, respectively. In addition, one of the novel miRNAs, m0246, was found to cleave as many as 38 target genes (S9 Table). One target gene of novel miRNAs, ppa006634m, was found to be cleaved by eight novel miRNAs (m0023, m0107, m0108, m0122, m0131, m0132, m0148, and m0149) at nucleotide 1252 of its sequence from the 5’ to the 3’ end. Three pairs of novel miRNAs (m0107 and m0108, m0148 and m0149, and m0091 and m0126) have the same sequences. m0023, m0122, m0131, and m0132 also have the same miRNA sequences but are located on different scaffolds or different positions of the same scaffold (S4 and S9 Tables). The same finding was also observed for m0028, m0103, and m0184. For the other target genes of known and novel miRNAs, no cleavage fragments or cleavage sites were detected through sequencing. In all, we could not detect the cleavage sites for most of the known miRNAs and newly predicted novel miRNAs in this degradome library. The degradome library sequencing data are available at NCBI-GEO under Accession No. GSE83110.

### qRT-PCR analysis of conserved and novel miRNAs

**Fig 4:**
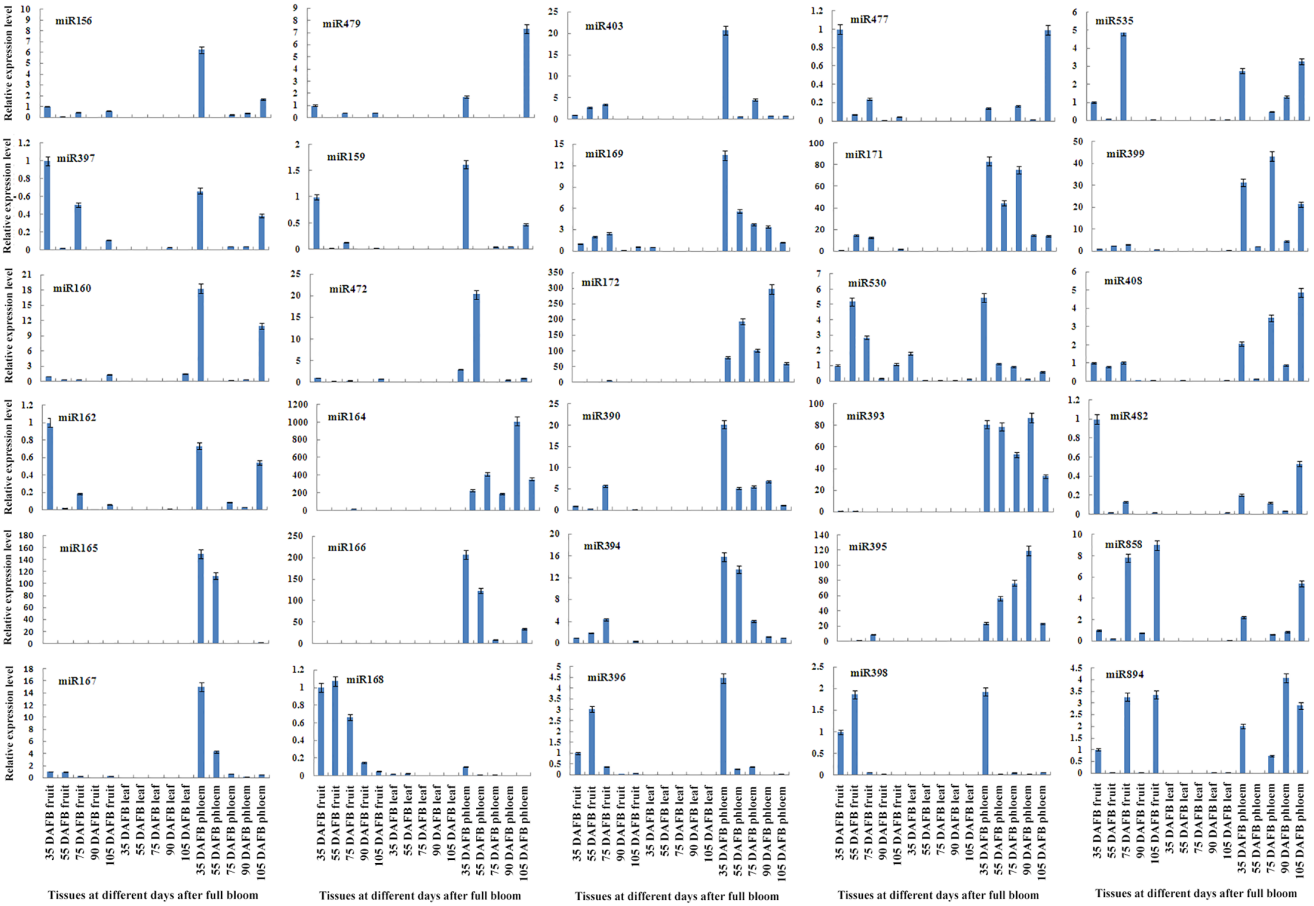
Relative expression levels of some known miRNAs in peach. The relative expression levels of known miRNAs in different tissues at five developmental stages were investigated by qRT-PCR analysis and calculated using the 2^-ΔΔCT^ method. The x-axes show the leaf, fruit, and phloem samples obtained 35, 55, 75, 90, and 105 days after full bloom (DAFB). The vertical bars represent the ±SE of each mean value (n = 9).

**Fig 5:**
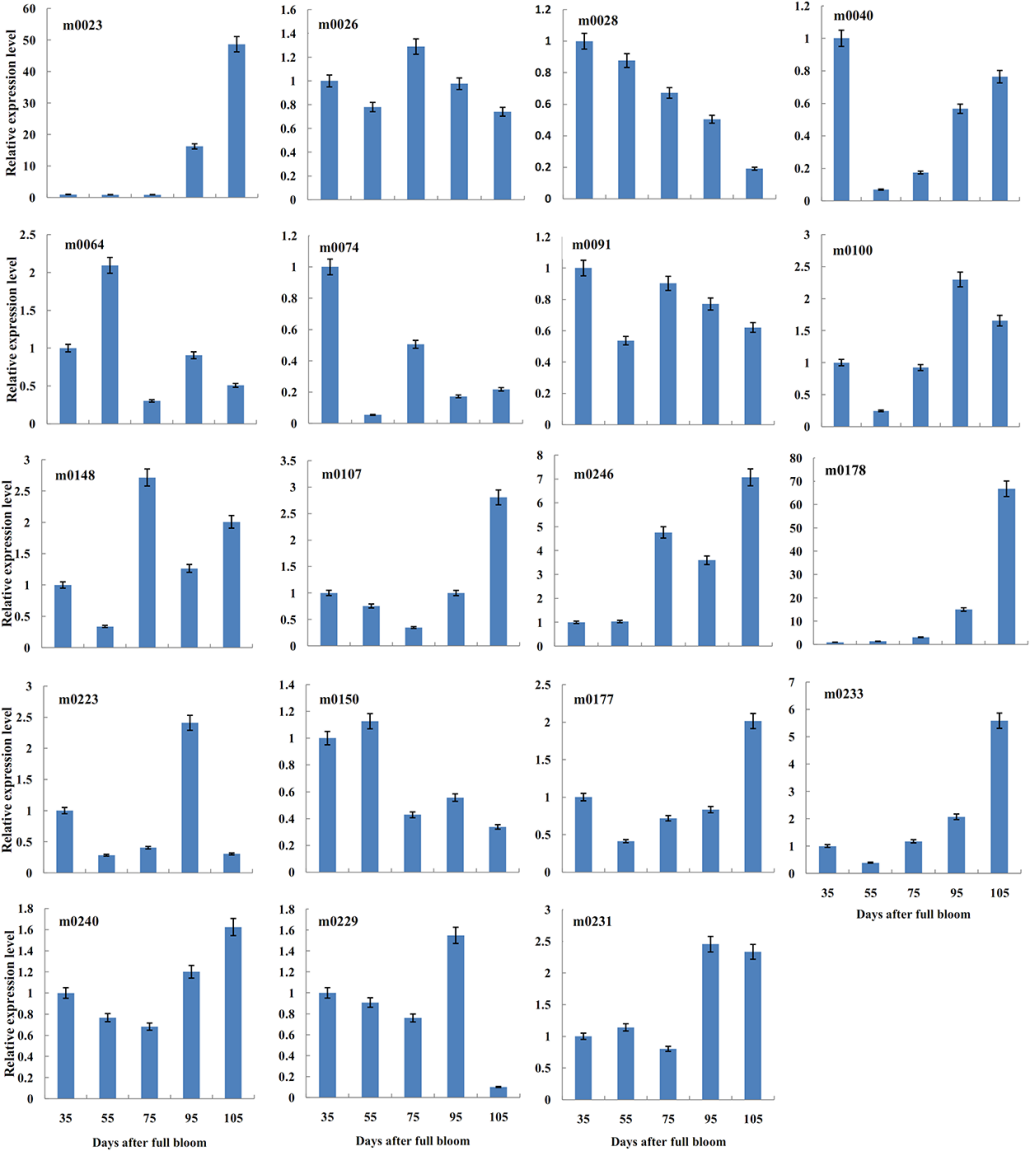
Relative expression levels of some novel miRNAs in peach fruit. The relative expression levels of novel miRNAs in fruit tissue at five developmental stages were investigated by qRT-PCR and calculated using the 2^-ΔΔCT^ method. The x-axes show the fruit samples obtained 35, 55, 75, 90, and 105 days after full bloom. The vertical bars represent the ±SE of each mean value (n = 9).

The spatiotemporal expression of miRNAs provides clues regarding their physiological functions as well as fundamental evidence to support the existence of miRNAs in a given species. Of the 557 known miRNAs, some conserved miRNAs were selected, and a qRT-PCR analysis was performed to investigate the expression patterns of these known miRNAs in the peach leaf, phloem and fruit at different stages of development (Fig 4). The expression features of some miRNAs appeared to be specific to the development stage. miR164, miR165, miR166, miR172 and miR393 exhibited a similar phloem-specific expression pattern, i.e., they were highly expressed only in phloem tissues, whereas their expression level was relatively undetectable or very weak in the leaves and fruit at different stages. miR168 presented fruit-specific expression because it was highly expressed but decreased with progressing development. Most of the remaining miRNAs displayed varied expression levels in the different tissues at the different stages of development. Of the novel miRNAs with the same sequences but located on different scaffolds, we only selected one for the expression analysis. Thus, the relative expression levels of 19 out of 27 novel miRNAs in fruit tissue were detected in this study (Fig 5). m0023 presented relatively equal expression levels from 35 to 75 days and then presented a rapid increase in expression. m0178 showed an obvious increasing trend during fruit development until maturity. On the contrary, m0028 displayed a decreasing tendency during fruit development. m0040, m0107, m0233, and m0240 showed high expression levels in the youngest fruit and then presented a decreasing trend as the fruit underwent development. m0026 and m0091 showed the same trend in terms of expression levels throughout the developmental period, whereas other miRNAs showed no specific tendency in this cultivar.

## Discussion

In recent years, several approaches have been developed for the identification of plant miRNAs. The Solexa platform employs a new high-throughput sequencing technology [42] and can generate a great number of sRNA sequence reads that are moderate in size compared with those of miRNAs. This platform combined with bioinformatics strategies has been widely used to identify miRNAs in various plants, including grapes [40], peanuts [21] and trifoliate orange [34]. Despite the importance of peach as an excellent model organism used to generate research information for the Rosaceae family, only a few miRNAs from the peach fruit sRNA library have been predicted. In addition to the identification of peach fruit miRNAs, this study provides a comprehensive analysis of peach miRNAs and their targets and reinforces the notion that the miRNAs in peach fruit are extremely complex and demonstrates that further exploration of the mechanisms through which each miRNA regulates its target genes and thus play different roles in each pathway is warranted.

Our study reveals a rich selection of sRNAs in peach fruit. The length distribution (Fig 1) of peach sRNAs resembles that in many other plants for which complete genome sequences are available, such as grapevine and rice [40,43]. The sRNA library assembled in this research study is of remarkably high quality compared with that of other sRNA libraries previously constructed for peach and other plant species (S10 Table). A considerably increased number of clean reads were produced from the peach fruit sRNA library compared with those obtained from many libraries of the other plant species despite the fact that peach has a genome of 220 to 230 Mb [44], which is shorter than that of *Oryza sativa* (420 Mb) [45] and grapevine (475 Mb) [46]. This observation confirms that woody plants (olive [47], grapevine [33], citrus [34], peach [22,23,25,48], and *Paulownia tomentosa* [49]) potentially have more clean reads than herbaceous plants (peanut [21], *Medicago truncatula* [50], and *Brassica juncea* [51]) (S10 Table).

Many plant miRNAs are evolutionarily conserved between species within the plant kingdom [52]. A typical conserved miRNA family contains several mature miRNAs with identical or near-identical sequences in different plant species. These provide a powerful base for predicting the sequences of orthologous miRNAs in other plant species. This property has enabled the identification of a large number of plant miRNAs with some being widely studied and many postulated to be conserved [7]. In this study, a majority of the known miRNA families in peach have also been identified in other species, such as *A*. *thaliana*, [53], rice [43], *B*. *napus* [54], maize [8], potato [55], *P*. *vulgaris* [20], and *Brachypodium distachyon* [56]. Axtell and Bartel [57] reported that the miR159/319 family exists in 10 plant species, whereas miR156 has been identified in 31 different types of plants, and approximately 30% of miRNA families examined, exist in at least 10 different types of plant species. In general, among the known miRNAs, evolutionarily conserved miRNAs exhibited a higher number of sequences (sequencing frequency) than unconserved miRNAs, as demonstrated in the current as well as previous studies [6]. miR156, miR157, and miR166 are the three most abundant known miRNAs in the peach fruit library and are also found in abundance in most plant species [23,58] (S2 Table). miRNA conservation indicates that plant miRNAs have a very deep origin in plant phylogeny, at least since the last common ancestor of bryophytes and seed plants [59].

In addition to the prediction of known miRNAs, a total of 275 novel miRNAs were predicted based on their biogenesis and the criterion of the stem-loop structure of potential pre-miRNAs. All the 275 miRNA precursors could be folded by RNAfold software into the hairpin structure and thus met the established criteria of miRNA annotation [35]. The total number of novel miRNAs was less than that of the known miRNAs. The absolute sequencing frequencies of the novel miRNAs were also considerably reduced, with most of the miRNAs having only several or dozens of copies, with the exception of m0187 and m0189, which were sequenced 6973 and 5552 times, respectively (S2 and S4 Tables). This observation was in agreement with previously reported results [36–38], which show that a majority of species-specific novel miRNAs present relatively lower sequencing frequencies and that their expression is considerably more spatiotemporal in nature than that of their conserved counterparts. Consistent with previous studies in other plant species [60], a large percentage of miRNAs starting with a U nucleotide at the 5’ end was also observed in the peach fruit sRNA library. This finding may be attributed to the key function of uracil in the recognition of miRNAs by AGRONAUTE1 [60].

To shed light on the putative functions of miRNAs in a plant species/tissue/stage, the initial prediction of target genes is necessary. Currently, bioinformatics analysis based on high complementarity between target genes and miRNAs is considered the most efficient and favored approach for predicting the target genes of miRNAs [61, 62]. In this study, higher numbers of targets for both the known and the novel miRNAs were predicted through a bioinformatics approach, and the targets and miRNAs were aligned based on their best orthologs in *A*. *thaliana* and other plants. A majority of the predicted target genes of conserved miRNAs in peach fruit encode the same family of transcription factors with miRNA target genes in *A*. *thaliana*, and these miRNA target sequences are also highly conserved across a wide range of plant species, as indicated by Floyd and Bowman [59]. Eleven out of 16 targets of miR156 identified in this study are transcription factors of the squamosa promoter-binding protein-like (SPL) family (S5 Table), which is also named the SBP family in some reports [55,60]. Similar results have been reported for potato [55], *Aquilegia coerulea* [60], and peach fruit [22], indicating that most targets of miR156 are transcription factors of the SPL family. Transcription factors of the SPL family have been reported to play an important role in regulating the floral meristem identity gene *squamosa1* [63]. In this study, one of the target proteins of miR156, SPL9 (GO: 2000027, ppa021582m) was annotated to function in the regulation of organ morphogenesis and to be involved in organ development, the regulation of organ morphogenesis, reproduction, system development, the hormone-mediated signaling pathway, and shoot system development (S6 Table). SPL3 (ppa012607m), SPL4 (ppa011968m), SPL6 (ppa003644m), SPL9 (ppa007056m), and SPL10 (ppa024285m) were annotated to have the same unique function of nucleic acid binding (S6 Table). However, the remaining five targets of miR156 predicted in this study encode different transcription factors than those found in potato and *A*. *coerulea* [55,60] (S5 Table). The total number of targets of miR156 predicted in this study varied from that found in other plant species. These observations indicate that known miRNAs of peach fruit contain targets with complementary binding sites that are highly conserved in other plants and have also evolved new targets to expand their regulatory functions in peach fruit. This expansion of functions may be due to the need to support features of physiology and reproduction in peach fruit.

Consistent with earlier findings on novel miRNAs in *A*. *thaliana* and other plant species [53,59], a majority of the predicted target genes for novel miRNAs identified in this work encode various plant-specific transcription factors, such as NPR1 (nonexpressor of PR genes), NAM, SBP, and serine incorporator (Serinc superfamily). Additionally, various predicted target genes are associated with stress responses, ethylene responses, signal pathways, secondary metabolite biosynthesis, transport, and catabolism (S8 Table). Four out of five target genes of m0126, m0157, and m0260 encode proteins of the ARF family, ethylene-responsive transcription factor, and proteasome subunit beta type-4, respectively. We also discovered important target genes involved in carotenoid and carbohydrate metabolism for some peach fruit-specific miRNAs, including ppa021157m (carotenoid isomerase) and ppa007937m (aldose 1-epimerase), which were predicted to be targeted by the novel miRNAs m0245 and m0041, respectively. This finding is important for peach breeding and calls for additional and much more intensive research on these miRNA target genes to obtain a greater understanding of the mechanisms involved in the development of flavor in peach fruit. Additionally, the novel m0233 (CTGACAGAAGAGAGTGAGCAC, S4 and S8 Tables) has the same 11 target genes encoding transcription factors of the SPL family as the known miR156 (TTGACAGAAGAAAGAGAGCAC, S2 and S5 Tables). Thus, we proposed that one target might simultaneously be regulated by both novel and conserved miRNAs in peach fruit. Similarly, one target gene targeted by several conserved miRNAs was also found in this study (S5 Table). Interestingly, three target genes (ppa012437m, ppa013211m, and ppa017834m) were all found to be targeted by 13 novel miRNAs that had precisely the same miRNA sequence (AAAGACTAAAATACCCTTGA) but at different locations and scaffolds (S4 and S8 Tables).

To date, no miRNA target in peach fruit has been identified by 5’RACE or degradome sequencing. The results of the degradome sequencing performed in this study illustrate that degradome sequencing is an efficient method for validating the targets of miRNAs and the cleavage sites. The degradome sequencing results confirmed that 115 and 101 target genes were cleaved by 60 known miRNA families and 27 novel miRNAs identified in this study, respectively (S9 Table). This result provides a reliable basis for the following further studies. In contrast, the cleavage fragments or sites for the other 3844 of the total 3959 predicted target genes of known miRNAs and 1513 of the 1614 predicted target genes of novel miRNAs were not detectable through degradome sequencing, suggesting that these target genes are likely regulated by miRNAs through translational repression rather than cleavage [64]. This finding is consistent with previous deductions that the translational regulation of target genes by miRNAs may be more important than cleavage [16]. Alternatively, it is possible that these target genes are not regulated by miRNAs in these growth stages or that these miRNAs may function in other specific developmental stages or environments that have not yet been analyzed. Therefore, a number of further studies are needed to obtain a more in-depth understanding of these miRNAs and their targets.

Consistent with findings in other plants [25,65], we found that the known miRNA-directed cleavage of targets was biased toward conserved miRNA families in peach fruit. Twenty-one conserved miRNA families of the verified 60 known miRNA families were found to cleave 54 target genes, which means that one conserved miRNA family cleaves an average of 2.57 target genes. However, the remaining 39 non-conserved miRNA families of the 60 known miRNA families were found to cleave the remaining 61 target genes, which means that each non-conserved miRNA family only cleaves an average of 1.56 target genes (S3 and S9 Tables). This finding leads to the hypothesis that one conserved miRNA may not only have a conserved regulation mechanism in terms of common features of morphological and physiological processes throughout the plant kingdom but also act induce signal transduction in the regulation of multi-target networks in one tissue or stage of a plant [65].

The miR166 family is widely considered to have highly conserved targets that encode proteins of the HD-ZIP family in a wide range of plant species, such as olive [47], citrus [62], and cotton [32]. This protein family regulates critical aspects of plant development, including lateral organ polarity, apical and lateral meristem formation, and vascular development [66]. Remarkably, we found that miR166 in peach fruit has an identical miRNA sequence, the same target gene number, the same miRNA-binding sites, and identical cleavage sites as those of miR166 in *Arabidopsis* [67]. Additionally, the four target genes of miR166 in peach also have a high level of sequence homology with those in *Arabidopsis* [67]. Consistent with earlier studies in plants [68], this finding reveals that the coevolution of miRNAs and their targets plays an important role in the conservation of gene regulation mechanisms and results in similar morphological or physiological features. In contrast, this finding verifies that the miRNA systems of plants have many features in common with the miRNA systems of animals [69].

The spatiotemporal expression of miRNAs in different tissues or developmental stages of the tissue may provide clues regarding their roles in phenotype and physiological functions. A qRT-PCR expression analysis of conserved miRNAs in three tissues at diverse developmental stages revealed that most of the conserved miRNAs appeared to be most strongly expressed in the peach fruit or phloem at the particular developmental stages studied (Fig 4). With the exception of miR169 and miR530, the expression levels of the other conserved miRNAs were found to be very low or even almost undetectable in the leaves compared with the levels in the fruit and phloem tissues. This finding suggests that conserved miRNAs exhibit a tissue-specific pattern. Additionally, the majority of the conserved miRNAs studied in this work present incoherent expression patterns at different stages in the peach fruit or phloem, implying that these miRNAs also present development-stage-specific expression patterns. In part, these expression results verify a previous finding that the stability of miRNAs, which depends on the stage of development or cell type involved, importantly determines the miRNA levels in the cell [70]. The specific expression patterns detected in this study for some miRNAs in peach fruit note the functional specificity of these miRNAs during development periods. This finding raises interesting questions that should be emphasized in future studies given the importance attached to peach fruit. Family expansion induces genes to generate novel miRNAs with species-specific features [71]. In this study, 19 novel miRNAs showed differential expression levels in peach fruit at five different developmental stages (from young to mature fruits) (Fig 5). Among these, m0028 and m0178 exhibited gradual decreases and increases during the fruit developmental process, respectively. According to the degradome sequencing results obtained in this study, m0028 was verified to cleave a transcription factor of the GRAS family, which is reported to function in the activation of meiosis-related gene transcription and was found to be involved in the regulation of microsporogenesis in the lily anther [72]. The combination of these functions of the GRAS family of transcription factors with the negative regulation of target genes by miRNAs suggests that m0028 may be associated with fruit proliferation and regulate cell meiosis to increase fruit size during fruit development. This conclusion is consistent with the findings in grape, *Prunus mum*, and tomato, which propose that *GRAS* family genes are associated with the cell cycle, cell growth and differentiation and the control of fruit development at different stages [73–75]. The majority of novel miRNAs do not exhibit specific trends with respect to their expression levels at the five studied developmental stages. This finding may be due to the fact that multiple factors can regulate miRNA expression in plants [76]. Some of these include transcription, processing, biogenesis, and RISC incorporation, which can collectively determine the miRNA levels in a tissue and at a stage [12]. Furthermore, the environmental conditions at different sampling dates, such as heat, ozone, and drought, also affect miRNA expression [77].

The findings of this study provide a basis for the future analysis of miRNA functions in peach fruit. Further studies should be designed and executed to provide a more comprehensive understanding of these miRNAs and the functions of their targets.

## Supporting Information

S1 Table Primer sequences of selected miRNAs used for qRT-PCR analysis. (DOCX)

S2 Table Summary of known miRNAs identified by high-throughput sequencing in a library of the small RNAs of peach fruit. The summary includes the length, count, and score of each miRNA, the best homolog of each peach miRNA in other plant species, and the number of matches and mismatches between the best homolog in other plant species and the peach miRNA.
(XLSX)

S3 Table Summary of homologs of conserved miRNA families identified in a library of the small RNAs of peach fruit. Double plus symbols (++) indicate that the miRNA sequences of peach fruit are exactly identical to those in other species. Single plus symbols (+) indicate that the miRNA sequences of peach fruit are conserved in other species but exhibit variations in some nucleotide positions.
(DOCX)

S4 Table Summary of novel miRNAs and alignment of miRNAs to their pre-miRNAs predicted from a library of the small RNAs of peach fruit. The summary includes the chromosome location, the start and end positions on the chromosome of each miRNA sequence, the minimum free energy, the miRNA orientation, the pre-miRNA sequence, the structure, the miRNA sequence, and the alignment of each miRNA to its pre-miRNA.
(XLSX)

S5 Table Targets of known miRNAs, targeted proteins, and alignment of each miRNA (top sequence) to the target site (bottom sequence) in a library of the small RNAs of peach fruit. (XLSX)

S6 Table GO annotation analysis of target genes of known miRNAs in a library of the small RNAs of peach fruit. (XLSX)

S7 Table KEGG pathway analysis of target genes of known miRNAs in a library of the small RNAs of peach fruit. (XLSX)

S8 Table Targets of novel miRNAs and alignment of each miRNA (top sequence) to the target site (bottom sequence) in a library of the small RNAs of peach fruit. (XLSX)

S9 Table Identified miRNAs, targets, and cleavage sites in peach fruit obtained by degradome sequencing. (XLSX)

S10 Table Number-based comparison of small RNA reads and miRNAs in different plants obtained by sequencing. (DOCX)

## Abbreviations

ARF family: auxin response factor family
DABF: days after full bloom
GDR: Genome Database for Rosaceae
GO: gene ontology
HD-ZIP protein: homeodomain-leucine zipper protein
KEGG: Kyoto Encyclopedia of Genes and Genomes
MFE: minimum free energy
miRNA: microRNA
NAM family: no apical meristem Family
NCS1 family: nucleobase cation symporter-1 family; nt: nucleotide
qRT-PCR: quantitative reverse transcription-polymerase chain reaction
SOAP: Short Oligonucleotide Alignment Program
sRNA: small RNA
TT2 family: transparent testa 2 family
U: uridine
