# Supplementary material for: Identification of Known and Novel microRNAs and Their Targets in Peach (Prunus persica) Fruit by High-Throughput Sequencing
Source: PLoS One. 2016 Jul 28;11(7):e0159253. doi: 10.1371/journal.pone.0159253 (PMC4965121; doi:10.1371/journal.pone.0159253)
Supplement: S1 Table — (DOCX) [file pone.0159253.s001.docx]

**S1 Table. Primer sequences of selected miRNAs used for qRT–PCR analysis**

| miRNA | Primer sequence (5'-3') | miRNA | Primer sequence (5'-3') |
| --- | --- | --- | --- |
| miR156 | TTGACAGAAGAAAGAGAGCAC | miR390 | AAGCTCAGGAGGGATAGCGCC |
| miR479 | TGTGATATTGGTTCGGTTCAT | miR393 | TCCAAAGGGATCGCATTGATC |
| miR397 | TCATTGAGTGCAGCGTTGATG | miR394 | TTGGCATTCTGTCCACCTCC |
| miR159 | TTTGGATTGAAGGGAGCTCTA | miR395 | CTGAAGTGTTTGGGGGAACTC |
| miR160 | GCGTACGAGGAGCCAAGCATA | miR396 | TTCCACAGCTTTCTTGAACTG |
| miR472 | TCTTTCCCAATCCACCCATGCC | miR398 | TGTGTTCTCAGGTCGCCCCTG |
| miR162 | TCGATAAACCTCTGCATCCAG | miR399 | CTGCCAAAGGAGATCTGCTCAG |
| miR164 | TGGAGAAGCAGGGCACGTGCA | miR403 | TTAGATTCACGCACAAACTCG |
| miR165 | TCGGACCAGGCTTCATCCCCC | miR408 | ACAGGGAACAGGTAGAGCATG |
| miR166 | TCGGACCAGGCTTCATTCCCC | miR477 | ACTCTCCCTCAAAGGCTTCTAG |
| miR167 | TGAAGCTGCCAGCATGATCTA | miR482 | GGAATGGGCTGTTTGGGATG |
| miR168 | TCGCTTGGTGCAGGTCGGGAA | miR535 | TGACGACGAGAGAGAGCACGC |
| miR169 | TGAGCCAAGAATGACTTGCTG | miR858 | TTCGTTGTCTGTTCGACCTGA |
| miR171 | CGAGCCGAATCAATATCACTC | miR894 | GTTTCACGTCGGGTTCACCA |
| miR172 | AGAATCTTGATGATGCTGCAT | 5S rRNA-F | CTCGGCAACGGATATCTCGGCTCT |
| miR530 | TGCATTTGCACCTGCACTTGT | 5S rRNA-R | CTAATGGCTTGGGGCGCAACTTG |
| m0023 | CAGCCAAGGATGACTTGCCGG | m0150 | TATCCAAGTAGAGAACTTAAA |
| m0026 | TTGCCTATTCCTCCCATGCCAA | m0177 | GGGCGTCTCTCCATTGGCAG |
| m0028 | TGATTGAGCCGTGCCAATATC | m0178 | CAGTGGAAGTAGCAAGGGGAA |
| m0040 | TCCATCGGAGTTACTATTCATC | m0223 | AAGCTGCCAACATGTTCGTCT |
| m0064 | TATGGCAGGAAAGAATGTGA | m0229 | TCCAGGATTCTCGGGTCATAG |
| m0074 | TTCTTTCCTCTTTCGTTTCCA | m0231 | TTGAGCCGCGCCAATATCACT |
| m0091 | TGCCTGGCTCCCTGTATGCCA | m0233 | CTGACAGAAGAGAGTGAGCAC |
| m0100 | TTCCCAAGCCCGCCCATTCCAA | m0240 | AGAGGCGGAGAGAGAGAGAGACA |
| m0107 | TAGCCAAGGATGACTTGCCT | m0246 | AGAGACAGAGAGAGACAGAGACA |
| m0148 | GAGCCAAGGATGAATTGCCGG |  |  |
