# Supplementary material for: Identification of Known and Novel microRNAs and Their Targets in Peach (Prunus persica) Fruit by High-Throughput Sequencing
Source: PLoS One. 2016 Jul 28;11(7):e0159253. doi: 10.1371/journal.pone.0159253 (PMC4965121; doi:10.1371/journal.pone.0159253)
Supplement: S3 Table — Double plus symbols (++) indicate that the miRNA sequences of peach fruit are exactly identical to those in other species. Single plus symbols (+) indicate that the miRNA sequences of peach fruit are conserved in other species but exhibit variations in some nucleotide positions. (DOCX) [file pone.0159253.s003.docx]

**S3 Table. Summary of homologs of conserved miRNAs identified in a library of the small RNAs of peach** **fruit**. Douple two plus symbols (++) indicate that the miRNA sequences of peach fruit are exactly identical to those in other species. Single plus symbols (+) indicate that the miRNA sequences of peach fruit are conserved in other species but exhibit variations in some nucleotide positions.

| miRNA family | Length (nt) | Sequence (5’-3’) | Best candidate homolog | Mismatch number | Other homologs | | | | | |
| --- | --- | --- | --- | --- | --- | --- | --- | --- | --- | --- |
|  |  |  |  |  | ath | ptc | vvi | osa | bna | Other plants |
| miR156 | 21 | UUGACAGAAGAAAGAGAGCAC | smo-miR156c | 0 | + | + | + | + |  | ahy + |
| miR159 | 21 | UUUGGAUUGAAGGGAGCUCUA | ath-miR159a | 0 | ++ | ++ | + | + | ++ | ahy ++ |
| miR160 | 21 | UGCCUGGCUCCCUGUAUGCCA | ath-miR160a | 0 | ++ | ++ | + | ++ |  | ahy ++, smo++, zma + |
| miR162 | 21 | UCGAUAAACCUCUGCAUCCAG | ath-miR162a | 0 | ++ | ++ |  | ++ |  | ahy ++, zma + |
| miR164 | 21 | UGGAGAAGCAGGGCACGUGCA | ath-miR164a | 0 | ++ | ++ | ++ | ++ |  |  |
| miR165 | 21 | UCGGACCAGGCUUCAUCCCCC | ath-miR165a | 0 | ++ |  |  |  |  | Phaseolusvulgris + |
| miR166 | 21 | UCGGACCAGGCUUCAUUCCCC | ath-miR166a | 0 | ++ | ++ | + | ++ | ++ | zmo ++ |
| miR167 | 21 | UGAAGCUGCCAGCAUGAUCUA | ath-miR167a | 0 | ++ | ++ | + | ++ |  |  |
| miR168 | 21 | UCGCUUGGUGCAGGUCGGGAA | ath-miR168a | 0 | ++ | ++ |  | + |  | ahy ++ |
| miR169 | 21 | UGAGCCAAGAAUGACUUGCUG | ath-miR169a | 1 | + | + | + | + | + |  |
| miR171 | 21 | UUGAGCCGCGCCAAUAUCACU | vvi-miR171f | 0 |  | + | ++ | + | + |  |
| miR172 | 21 | AGAAUCUUGAUGAUGCUGCAU | ath-miR172a | 0 | ++ | ++ | + | ++ |  |  |
| miR390 | 21 | AAGCUCAGGAGGGAUAGCGCC | ath-miR390a | 0 | ++ | ++ |  |  | ++ |  |
| miR393 | 21 | UCCAAAGGGAUCGCAUUGAUC | osa-miR393 | 0 | + |  |  | ++ | + |  |
| miR394 | 20 | UUGGCAUUCUGUCCACCUCC | ath-miR394a | 0 | ++ | ++ | + |  | ++ |  |
| miR395 | 21 | CUGAAGUGUUUGGGGGAACUC | ath-miR395a | 0 | ++ | + | ++ | + |  |  |
| miR396 | 21 | UUCCACAGCUUUCUUGAACUG | ath-miR396a | 0 | ++ | ++ | + | ++ | + |  |
| miR397 | 21 | UCAUUGAGUGCAGCGUUGAUG | ath-miR397a | 0 | ++ | ++ | ++ | ++ | + |  |
| miR398 | 21 | UGUGUUCUCAGGUCGCCCCUG | osa-miR398b | 0 | + | ++ | ++ | ++ | + |  |
| miR399 | 22 | CUGCCAAAGGAGAUCUGCUCAG | osa-miR399e | 2 | + | + | + | + | + |  |
| miR403 | 21 | UUAGAUUCACGCACAAACUCG | ath-miR403 | 0 | ++ | ++ | ++ |  |  |  |
| miR408 | 21 | ACAGGGAACAGGUAGAGCAUG | mtr-miR408 | 2 |  |  |  |  |  |  |
| miR472 | 22 | UCUUUCCCAAUCCACCCAUGCC | ptc-miR472b | 1 |  | + |  |  |  |  |
| miR477 | 22 | ACUCUCCCUCAAAGGCUUCUAG | vvi-miR477 | 2 |  |  | + |  |  |  |
| miR479 | 21 | UGUGAUAUUGGUUCGGUUCAU | csi-miR479 | 1 |  | + | + |  |  |  |
| miR482 | 20 | GGAAUGGGCTGUUUGGGAUG | mdm-miR482* | 2 |  |  |  |  |  |  |
| miR530 | 21 | UGCAUUUGCACCUGCACUUGU | gma-miR530 | 0 |  | + |  | + |  |  |
| miR535 | 21 | UGACGACGAGAGAGAGCACGC | ppt-miR535a | 1 |  |  | + |  |  |  |
| miR823 | 21 | UGGGAUGGUGUAUAAUAAGAU | ath-miR823 | 0 | ++ |  |  |  |  |  |
| miR827 | 21 | UUAGAUGACCAUCAACAAACA | ghr-miR827a | 0 |  |  |  | + |  |  |
| miR858 | 21 | UUCGUUGUCUGUUCGACCUGA | ath-miR858b | 2 |  |  |  |  |  |  |
| miR894 | 20 | GUUUCACGUCGGGUUCACCA | ppt-miR894 | 0 |  |  |  |  | + |  |
| miR1447 | 21 | UCAGAAUUGCAGUGCCUUGAU | ptc-miR1447 | 0 |  | ++ |  |  |  |  |
| miR2111 | 20 | UAAUCUGCAUCCUGAGGUUU | ath-miR2111a | 0 |  |  |  |  |  |  |

Notes: ath, *Arabidopsis thaliana*; ptc, *Populus trichocarpa*; vvi, *Vitis vinifera*; osa, *Oryza sativa*; bna, *Brassica napus*; zma, *Zea mays*; ahy, *Arachis hypogaea*; pvu, *Phaseolus vulgaris*.
