# Supplementary material for: Identification of Known and Novel microRNAs and Their Targets in Peach (Prunus persica) Fruit by High-Throughput Sequencing
Source: PLoS One. 2016 Jul 28;11(7):e0159253. doi: 10.1371/journal.pone.0159253 (PMC4965121; doi:10.1371/journal.pone.0159253)
Supplement: S10 Table — (DOCX) [file pone.0159253.s010.docx]

**S10 Table. Number-based comparison of small RNA reads and miRNAs in different plants** **obtained by sequencing.**

|  |  | |  | | | | |  |  |  |
| --- | --- | --- | --- | --- | --- | --- | --- | --- | --- | --- |
| Plant species | Tissues sequenced | Clean reads  (total sRNA reads) | | Unique sRNAs | Redundant reads (total sRNA) matching its genome | Unique sRNAs matching the genome | Numbers of known miRNA | | Numbers of novel miRNA | Reference |
| *Brassica juncea* | Floral bud | 3,339, 269 | | - | 3, 192, 676 (95.61%) | - | 197 | | 78 | [51] |
| Peanut | Leaf, stem and root | 4,598,005 | | - | - | - | 75 | | 14 | [21] |
| Rice | Seedling | - | | - | 102,876 | - | 243 | | 23 | [43] |
| *Medicago truncatula* | Leaf | 3,948,871 | | 3,948,870 | 2,168,719 (54.92%) | 1,563,358 (39.59%) | - | | - | [50] |
| *C. trifoliata* | Flower and fruit | <13,106,573 | | 4,876,395 | - | - | 63 | | 10 | [34] |
| Grapevine | Flower and fruit | 16,231,320 | |  | 8,140,607 (50.15%) | 2,063,580 (38.05%) | - | | - | [33] |
| *Paulownia tomentosa* | Diploid leaf | 20,879,841 | | 9,399,611 | 17,147,888 (82.13%) | 7,256,739 (77.20%) | 35 | | 13 | [49] |
|  | Tetraploid leaf | 19,751,250 | | 8,525,686 | 16,347,595 (82.77%) | 6,522,175 (76.50%) | 37 | | 14 |  |
| Olive | Unripe fruit | 15,260,014 | | 7,933,475 | - | - | 136 | | 38 | **[47]** |
|  | Ripe fruit | 13,817,321 | | 7,423,620 | - | - | 136 | | 38 |  |
|  | Leaf, November, on year | 15,153,468 | | 6,003,166 | - | - | 136 | | 38 |  |
|  | Leaf, November, off year | 15,710,421 | | 6,001,443 | - | - | 136 | | 38 |  |
|  | Leaf, July, on year | 16,950,209 | | 5,479,750 | - | - | 136 | | 38 |  |
|  | Leaf, July, off year | 15,931,860 | | 5,535,758 | - | - | 136 | | 38 |  |
| Peach | Control leaf | 15,470,689 | | 4,210,911 | 10,264,244 (66.35%) | 2,063,684 (49.01%) | 531 | | 197 | [23] |
|  | Drought-stressed leaf | 12,428,654 | | 3,172,346 | 8,673,228 (69.78%) | 1,599,019 (50.40%) | 471 | | 221 |  |
|  | Control root | 12,539,747 | | 2,696,057 | 9,324,699 (74.36%) | 1,400,836 (51.96%) | 535 | | 238 |  |
|  | Drought- stressed root | 13,036,443 | | 4,125,069 | 8,157,867 (62.58%) | 1,747,201 (42.36%) | 487 | | 265 |  |
| Peach | Leaf | 10,151,770 | | - | - | - | 157 | | 230 | [22] |
|  | Winter bud | 10,899,501 | | - | - | - | 157 | | 230 |  |
| Peach | Root | 22,662,491 | | 4,365,343 | 19,376,109 (85.50%) | 2,955,635 (67.71%) | 47 | | 27 | [48] |
|  | Leaf | 18,295,552 | | 3,660,986 | 15,132,617 (82.71%) | 2,429,757 (66.37%) | 47 | | 27 |  |
|  | Flower | 5,128,828 | | 1,791,036 | 3,789,678 (73.89%) | 122,870 (6.90%) | 47 | | 31 |  |
|  | Fruit | 4,892,343 | | 834,891 | 3,472,776 (71.0%) | 453,500 (54.32%) | 47 | | 44 |  |
| Peach | Leaf,  stem and flower | 14,693,759 | | 4,259,405 | - | - | 117 | | 186 | [25] |
| Peach | Fruit | 16, 675, 173 | | 3,328,500 | 12,521,493 (75.09%) | 2,440,952 (73.33%) | 557 | | 193 | This work |

Note: ‘-’ indicates that data were not found.
